# Radiation-Induced Peripheral Artery Disease in a 63-Year-Old Patient

**DOI:** 10.7759/cureus.47372

**Published:** 2023-10-20

**Authors:** Claudia Aleman Oliva, Erik Aleman Espino, Michelle Demory Beckler, Marc M Kesselman

**Affiliations:** 1 Osteopathic Medicine, Nova Southeastern University Dr. Kiran C. Patel College of Osteopathic Medicine, Fort Lauderdale, USA; 2 Microbiology and Immunology, Nova Southeastern University Dr. Kiran C. Patel College of Allopathic Medicine, Fort Lauderdale, USA; 3 Rheumatology, Nova Southeastern University Dr. Kiran C. Patel College of Osteopathic Medicine, Fort Lauderdale, USA

**Keywords:** peripheral artery disease, postoperative radiation therapy, radiation-induced vascular damage, arterial damage, atherosclerosis, cardiovascular disease, case report, oxidative stress, cancer treatment side effects

## Abstract

Tobacco use, hypertension, diabetes, and hypercholesterolemia are known risk factors for peripheral artery disease (PAD). However, additional causes of PAD, such as radiation therapy, should be considered for the prevention and diagnosis of this disease. The patient described in this report had 36 radiation therapies directly to the pelvis and bladder area due to bladder cancer. The presence of severe PAD on this patient’s right external iliac artery, the same area where he received radiation therapy, raises the question of whether radiation therapy contributed to the development of PAD. In addition, his history of anal intraepithelial neoplasia, obstructive uropathy, and chronic kidney disease further demonstrated that he possibly suffered extensive tissue damage due to radiation to the pelvis. This case report explores the current diagnosis guidelines and treatment options for patients with radiation-induced PAD. Through this case study, we aim to bring awareness to this lesser-known cause of PAD among medical providers and promote research for the prevention and treatment of this disease.

## Introduction

Peripheral artery disease (PAD) affects approximately 7% of adults in the United States. The statistics show that PAD is more prevalent in older individuals and affects males and females equally. There is a higher incidence of PAD in patients with a history of tobacco use, hypertension, diabetes, and hypercholesterolemia [1]. A higher risk of PAD also exists in patients with chronic kidney disease (CKD). Spontaneous atherosclerosis is the most common cause of PAD; however, additional causes that may not be initially suspected include fibromuscular dysplasia, vasculitis, and radiation therapy for cancer that affects the pelvis and lower extremities, which can result in radiation-induced atherosclerosis [2,3]. The patient described in this report had 36 focused radiation therapies directly to the pelvis and bladder due to bladder cancer, which occurred 17 years before the diagnosis of PAD. He received the therapies five times per week for seven weeks.

Clinical presentation

The presentation of PAD ranges from asymptomatic to claudication, ischemic pain at rest, atypical pain, non-healing wounds, ulcerations, and gangrene. Usually, patients present with pain on exertion, and the severity of the disease can be determined by how long patients can walk before they experience pain [4].

Diagnosis

Risk factors, symptoms, physical exam findings, and ankle-brachial index (ABI) are used for the diagnosis of PAD. An ABI between 1.0 and 1.30 is considered normal, and an ABI of ≤0.90 is highly sensitive and specific for PAD [4]. In addition, the severity of the disease can also be assessed using ABI values. A resting or postexercise ABI of ≤0.9 indicates mild disease whereas an ABI of ≤0.70 (at rest) or ≤0.50 (postexercise) indicates moderate disease. Severe disease is associated with an ABI of ≤0.50, at rest, and <0.15, postexercise [2]. Vascular imaging is not necessary for diagnosis but might be indicated to differentiate PAD from other possible causes of arterial obstruction. Duplex ultrasound, computed tomography (CT) angiography, magnetic resonance angiography, and invasive contrast angiography can be used to localize the site of occlusion if intervention is being considered, but contrast angiography is the gold standard. Duplex ultrasound can also be used to monitor limb patency after revascularization [4]. When diagnosing patients with claudication, other possible etiologies should be considered, such as Baker’s cyst, venous claudication, chronic compartment syndrome, spinal stenosis, nerve root compression, and hip arthritis. In addition, screening for atherosclerosis in other vascular beds is recommended as patients with PAD have an increased risk of abdominal aortic aneurysm, and coronary, carotid, and renal artery atherosclerosis [5].

Management

The management of PAD focuses on slowing the progression of atherosclerosis, reducing the risk of adverse cardiovascular events, relieving symptoms, and preserving the functionality of the affected limb [2,6]. A supervised exercise program is the initial treatment for claudication, and it is highly effective [2,5]. Alternatively, low-intensity walking and cycling are also beneficial. Cilostazol is the drug of choice to treat symptoms of claudication and walking impairment [5]. Cilostazol is a phosphodiesterase III inhibitor (PDE3), which inhibits platelet aggregation and induces arterial vasodilation leading to increased blood flow to oxygen-deprived areas and improvement of claudication. Pharmacological treatments that reduce the progression of cardiovascular disease are also recommended and include long-term antithrombotic therapy with aspirin, clopidogrel, or ticagrelor. Clopidogrel and ticagrelor inhibit the P2Y12 receptor on platelets leading to failure of platelet activation and aggregation. P2Y12 inhibitors have been found to be more effective at reducing the risk of major adverse cardiovascular events (MACE) than aspirin. Recent studies have shown that the combination of aspirin and ticagrelor is beneficial in the reduction of both MACE and major adverse limb events (MALE) [7].

In addition, lipid-lowering therapy with well-established statins or the novel proprotein convertase subtilisin/kexin type 9 (PCKS9) inhibitor is recommended in PAD patients. Statins work by inhibiting the enzyme HMG-CoA reductase resulting in an increase in the expression of low-density lipoprotein (LDL) receptors in the liver and a reduction of LDL cholesterol in the blood. PCKS9 inhibitors (evolocumab and alirocumab) are monoclonal antibodies that reduce LDL levels and prevent atherosclerotic plaque formation by inhibiting inflammation and nuclear factor kappa B (NF-κB) activation. Statins and PCKS9 inhibitors have been associated with a lower risk of MACE and MALE in patients with PAD [7].

A newer alternative to statins is bempedoic acid, which inhibits adenosine triphosphate citrate lyase and has been shown to work similarly to statins by decreasing LDL levels. However, bempedoic acid has fewer of the muscular adverse effects related to statins, making it a good alternative for patients who do not tolerate the HMG-CoA reductase inhibitor [8]. Other emerging anti-atherosclerotic treatments include colchicine (a microtubule polymerization inhibitor), canakinumab (a monoclonal antibody that inhibits IL-1β), and ziltivekimab (a monoclonal antibody that inhibits IL-6) [9].

In addition to anti-atherosclerosis treatment, it is recommended to optimize the management of diabetes and hypertension if present in these patients. Lifestyle modifications such as smoking cessation have been shown to reduce these patients’ risk of cardiovascular events and limb loss. Revascularization is indicated in patients with persistent life-limiting claudication who do not respond to lifestyle modifications and pharmacologic treatment or in those with chronic limb-threatening ischemia. Revascularization can be attained through endovascular techniques (angioplasty with or without stenting) or surgical bypass grafting [5,6].

## Case presentation

**Figure 1:**
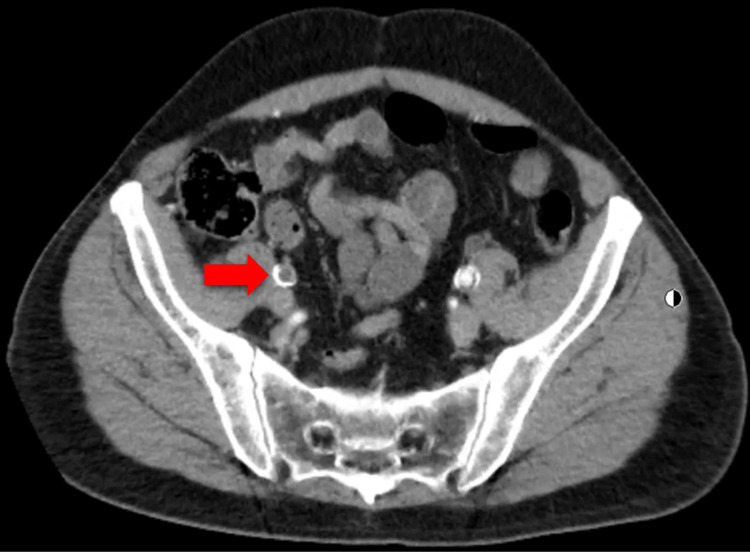
Abdominal pelvic computed tomography (CT) scan with contrast showing right external iliac artery occlusion. Red arrow, right external iliac artery occlusion.

**Figure 2:**
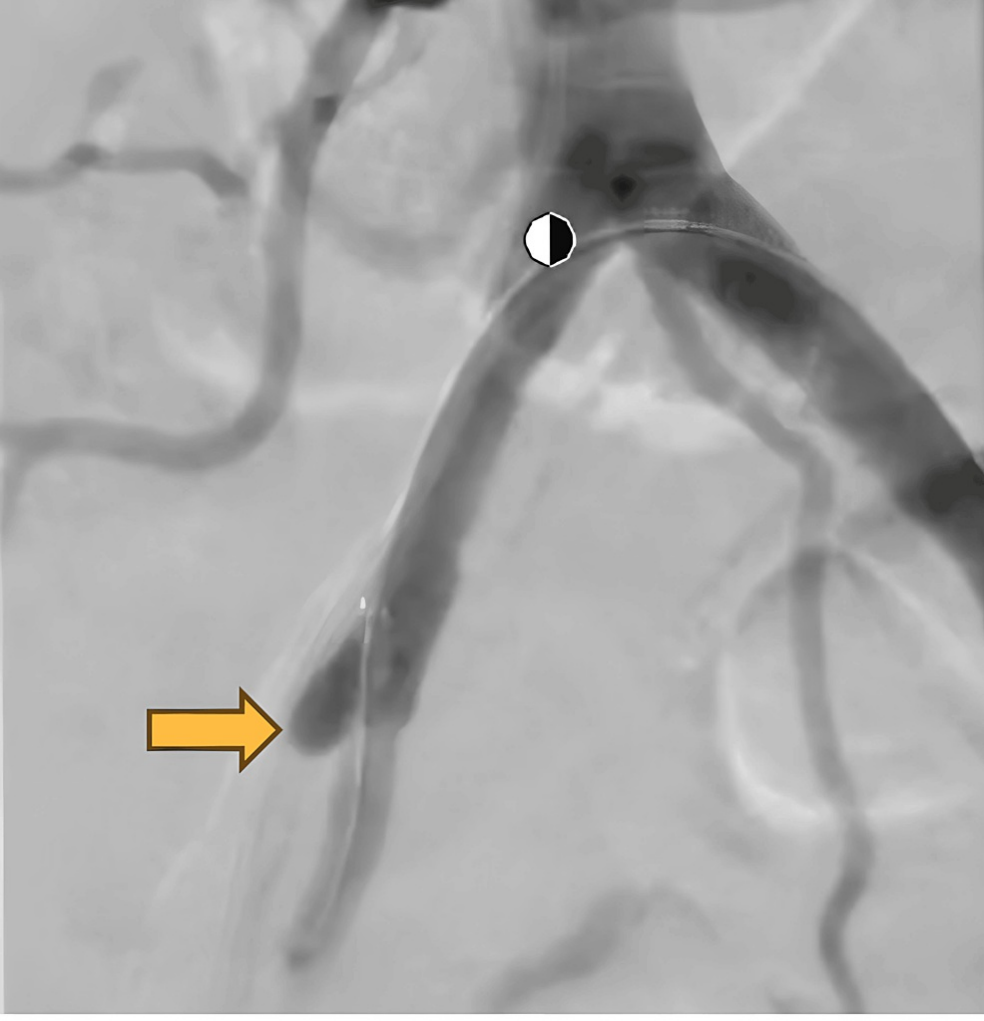
An angiogram showing occlusion of the right external iliac artery and a stump in the proximal external iliac artery. Yellow arrow, occlusion of right external iliac artery.

**Figure 3:**
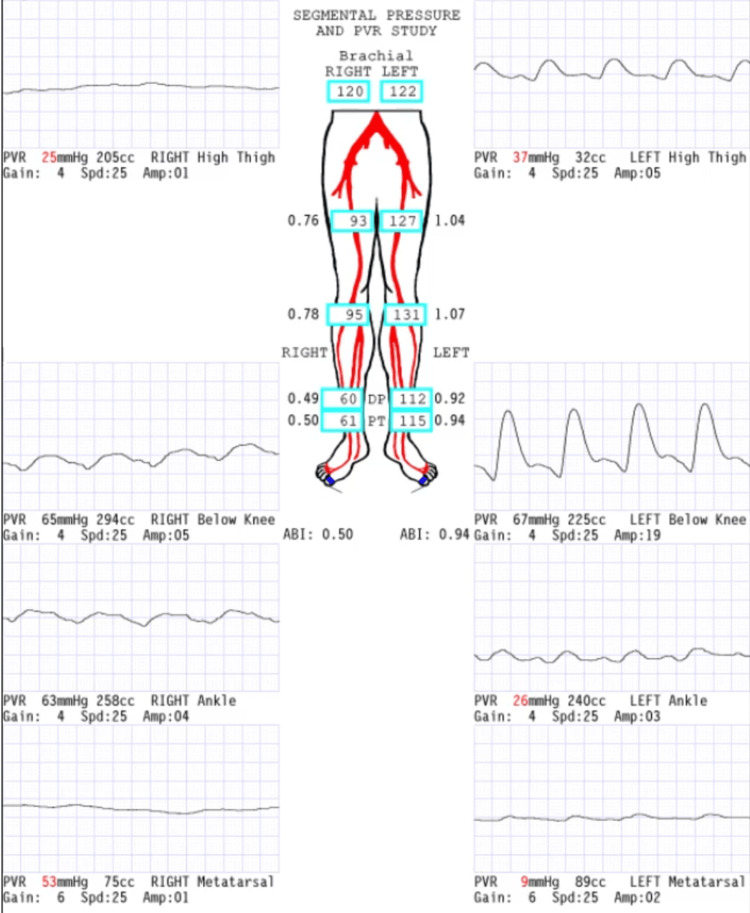
Segmental pressure and PVR study. PVR, pulse volume recording

A 63-year-old male presented to the primary care clinic for a routine visit. His past medical history included anal intraepithelial neoplasia (grade III), urothelial bladder carcinoma, CKD, combined hyperlipidemia, hypertension, and obstructive uropathy. His surgical history included bladder carcinoma resection (in 2003), laparoscopic cholecystectomy, and left laparoscopic nephrectomy (in 2021). The patient had a 30-pack-year history of tobacco use. An abdominal and pelvic CT scan with and without contrast was ordered by the Urology service and showed severe atherosclerotic disease in the lower abdominal aorta and its branches with occlusion of the right external iliac artery with reconstitution in the distal segment (Figure 1). During this initial visit, the patient denied claudication, chest pain, or muscle pain and was referred to the Vascular Surgery service. He was evaluated by a vascular surgeon one month after the primary care visit and reported life-limiting claudication of the right leg. The patient stated that the leg pain started after walking less than one block and severely interfered with activities of daily living. The patient was advised to undergo an angiogram with attempted canalization to improve symptoms. The procedure confirmed the occlusion of the right external iliac artery and a stump in the proximal external iliac artery (Figure 2). Revascularization was attempted, but vascular surgery decided against it because if a stent was placed at this location, it would occlude the origin of the right internal iliac artery. This could potentially lead to buttock claudication or pelvic ischemia; it was determined that the risks of revascularization outweighed the benefits, given the absence of limb-threatening ischemia. After the procedure, plethysmography ultrasound of the lower extremities was performed, which showed moderate-to-severe PAD on the right leg and mild PAD on the left leg. The ABI was 0.5 (normal range ABI = 1.0-1.30) on the right leg and 0.94 on the left leg on the segmental pressure and pulse volume recording (PVR) study (Figure 3). The patient was prescribed cilostazol 100 mg, a phosphodiesterase inhibitor, and aspirin 81 mg, and was advised to walk for 30 minutes every day. The patient continued with his previous treatment of omega 3, candesartan, rosuvastatin, and fenofibrate.

On a recent follow-up, the patient reported improvement since the initial visit, and he also reported that he could now walk a block and a half before the leg pain commences. The patient denied having any pain while resting, wounds on the lower extremities, or sensory loss. He reported that he continues to take cilostazol, aspirin, and rosuvastatin, but is inconsistent with exercise. Doppler ultrasound done in 2022 showed diffuse low peak systolic arterial velocities in both lower extremities, more prominent on the right leg, and minimal flow in the right dorsalis pedis. A low peak systolic arterial velocity is consistent with a monophasic waveform, indicating a more than 50% reduction in the blood vessel diameter due to an occlusion [10]. ABI at this time was 0.40 (normal range ABI = 1.0-1.30) on the right leg and 0.79 on the left leg. An evaluation of the carotid and coronary arteries has not yet been performed as of this report; however, the patient denies any overt symptoms that could indicate coronary artery disease or carotid artery stenosis. An assessment of these vascular beds is scheduled to be performed.

## Discussion

The presence of severe PAD on this patient’s right external iliac artery, the same area where he received external beam radiation therapy, raises the question of whether atherosclerosis might have been caused by radiation therapy. The patient had a medical history of hypertension and CKD, both well-known contributors to atherosclerosis. However, in patients, such as the one described here, who have a long history of compliance with medical treatment, atherosclerosis does not usually progress rapidly [11,12]. As a result, it is likely that the extra effect of the radiotherapy precipitated an increase and acceleration in the extent of the atherosclerotic disease seen in this patient.

Radiation therapy is not exclusively targeted to cancerous cells and can affect surrounding healthy tissue. The radiation field size is determined by the extent of the disease. Toxicity of treatment can carry significant morbidity and mortality if severe. Therefore, efforts to minimize radiation field size carry considerable importance in the treatment of cancer. In the case of this patient, the radiation field included the vasculature, which raised the risk of damage. The patient presented had previously been treated for anal intraepithelial neoplasia (grade III) and had a history of obstructive uropathy and CKD that led to a left kidney nephrectomy. The development of these conditions in the area where he received radiation further suggests that he possibly suffered extensive tissue damage due to this therapy. This is based on our knowledge that focal radiation can disrupt epidermal and vascular tissues, which can lead to an increase in inflammatory mediators and, subsequently, result in atherosclerosis [13,14].

Ionizing radiation therapy has been used to treat multiple types of cancers for more than a hundred years and has contributed to an increase in the survival of cancer patients. However, this increase in life expectancy has led to a higher incidence of radiation-induced cardiovascular disease. The damage caused by radiation therapy to the coronary arteries and the heart is well-established in the literature; however, the link between radiation and PAD has not been extensively investigated [3,13].

The mechanism of vessel injury associated with radiation-induced PAD is identical in all different vascular beds. Linear energy transfer is defined as the total energy that an ionizing particle places on a material as it goes across and is the foundation of radiation therapy. As ionizing particles penetrate healthy cells, reactive oxygen species (ROS) can form in the cytoplasm, which can lead to more ROS being released from the mitochondria [13]. The increase in ROS triggers the tyrosine phosphorylation and inactivation of IκB-α, which leads to the activation of NF-κB [14].

**Figure 4:**
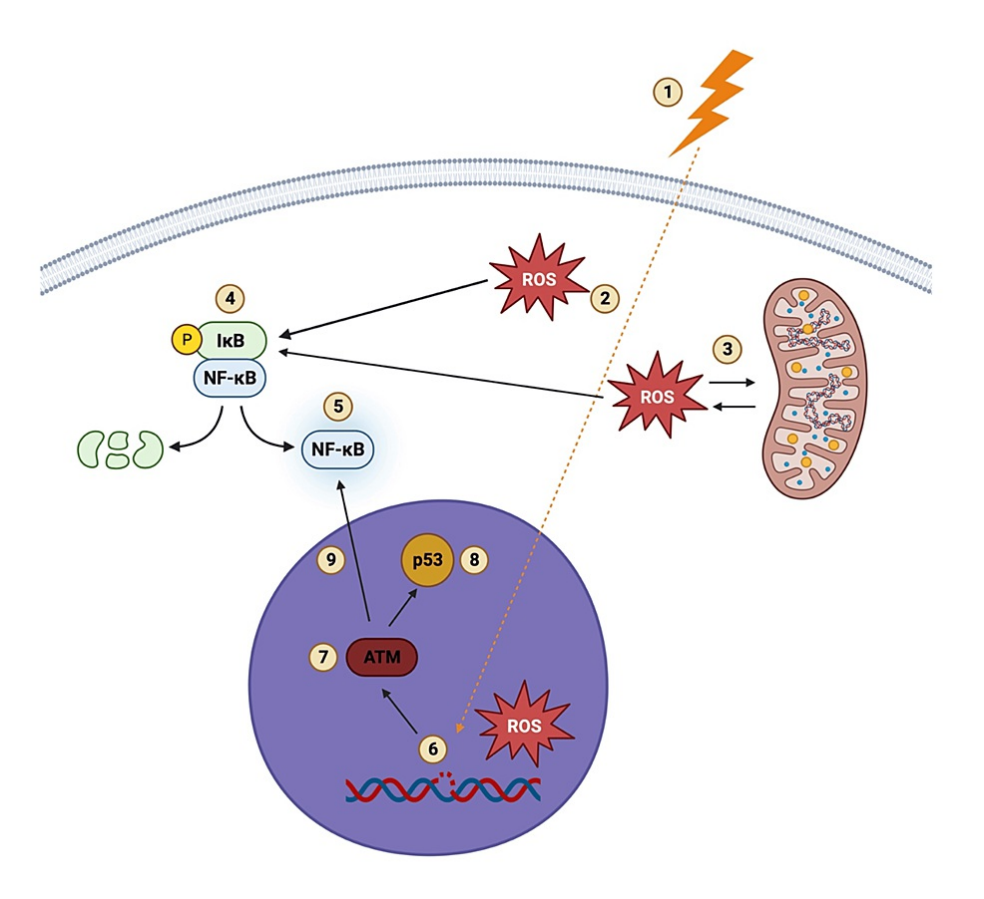
Mechanism of radiation-induced endothelial cell damage. (1) Ionizing particles penetrate healthy cells which leads to (2) the formation of ROS in the cytoplasm. (3) ROS in the cytoplasm induces ROS release from the mitochondria, and (4) the increased cytoplasmic ROS leads to the phosphorylation and inactivation of IκB-α, which results in (5) NF-κB activation. (6) In the nucleus, the formation of ROS and ssDNA breaks lead to (7) the activation of ATM and ATR (ATM and Rad3-related protein kinases) and (8) p53 activation which also results in (9) NF-κB activation [13,14,15]. ROS, reactive oxygen species; IκB-α, NF-κB inhibitor alpha; NF-κB, nuclear factor kappa B; ATM, ataxia telangiectasia mutated kinase; ssDNA, single-stranded DNA. Image credits: Created by the author using BioRender software (BioRender, Toronto)

Once in the nucleus, ionizing particles can lead to additional ROS formation and single-stranded DNA breaks and, consequently, activation of repair mechanisms such as the ataxia telangiectasia mutated kinase (ATM and Rad3-related kinase). These kinases can then phosphorylate a number of downstream targets, including the checkpoint proteins p53 and CHK kinases, arresting the cell cycle and activating NF-κB. The double-stranded DNA breaks are a major contributor to endothelial cell death, senescence, and an imbalance in the proliferation and differentiation of these cells, which leads to endothelial cell dysfunction. Senescent endothelial cells continue to produce ROS and inflammatory mediators (IL-1, IL-6, TNF-α, matrix metalloproteinases, and ICAM-1) through the activation of NF-κB in both the cytoplasm and nucleus (Figure 4) [13,15].

This results in a chronic inflammatory state that can persist for months after treatment, and it is characterized by the constant but unsuccessful remodeling of the endothelial cells. This leads to the recruitment of monocytes to the subendothelial space, where they turn into macrophages when exposed to high cholesterol levels in the blood. Subsequently, macrophages engulf cholesterol and become foam cells that produce fatty streaks in the tunica intima [14]. This process results in the accelerated form of atherosclerosis that is usually localized to the area affected by radiation therapy in younger individuals. Radiation to vasculature can also result in subintimal fibrinoid substance accumulation, smooth muscle degeneration, adventitia fibrosis, and vasa vasorum obliteration [3,13].

According to a recent article by the International Cardio-Oncology Society, early screening of PAD symptoms, pedal pulse assessment, and auscultation of renal and aortic arteries are recommended, and a review of CT scans is also recommended for possible arterial calcifications in patients with a history of radiation to the abdomen and pelvis [16]. The United States Preventive Services Task Force does not recommend the use of ABI to screen for PAD in asymptomatic patients [17]. However, screening with ABI before the onset of claudication might be beneficial in patients with radiation-induced PAD since these patients present with an accelerated form of atherosclerosis. 

**Figure 5:**
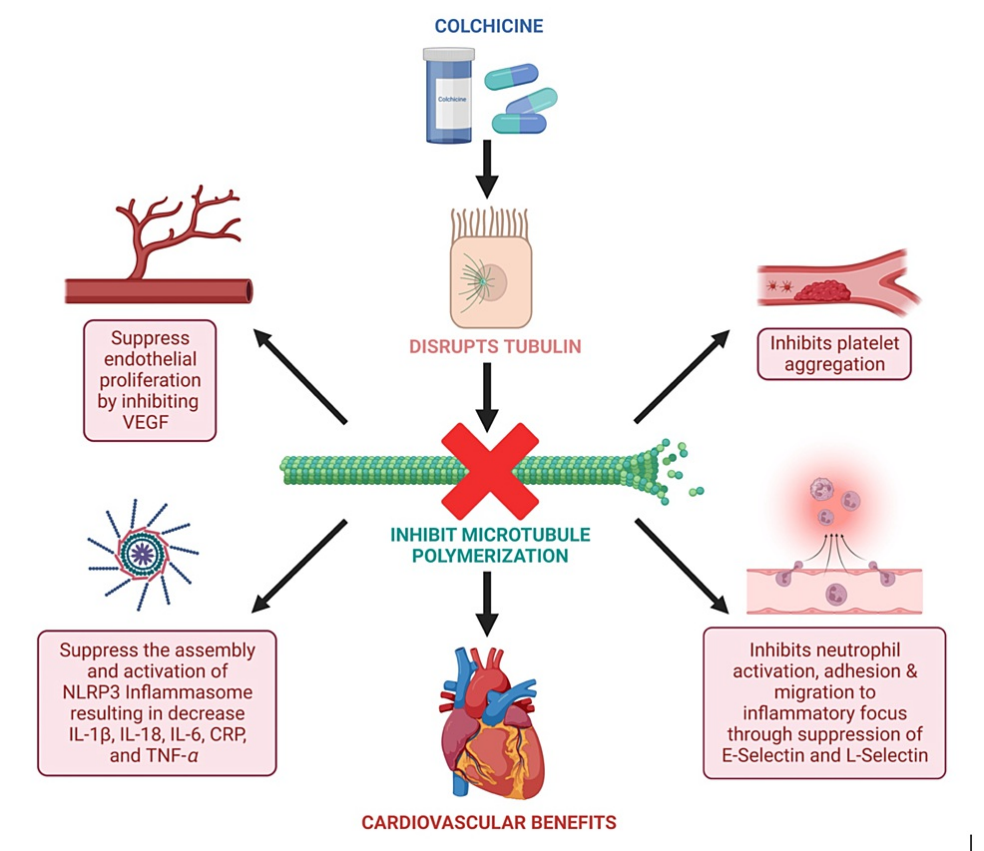
Mechanism of action of colchicine. VEGF, vascular endothelial growth factor; NLRP3, nucleotide-binding domain, leucine-rich–containing family, pyrin domain–containing-3; IL, interleukin, TNF-α, tumor necrosis factor-alpha; CRP, C-reactive protein. Image credits: Created by the author using BioRender software (BioRender, Toronto) Source: [20,21]

Currently, the recommended treatment for patients with radiation-induced PAD is the same as for the general population with PAD, and the only preventive measure is the reduction of atherogenic risk factors. Further research should investigate medications that can be used to prevent the early onset of PAD in these patients. Colchicine has been found to prevent radiation-induced coronary artery disease, which has the underlying mechanism of PAD [18]. Colchicine is a microtubule polymerization inhibitor, which binds to tubulin and inhibits tubulin polymerization, leading to the malfunction of the cell cytoskeleton, mitosis, and intracellular transport (Figure 5). This can result in an inhibition of platelet aggregation and a failure in neutrophil activation, adhesion, and migration to sites of inflammation due to a decrease in L-selectin and E-selectin [19]. In addition, colchicine suppresses the assembly and activation of the NLRP3 inflammasome, which leads to a decrease in inflammatory markers (IL-1β, IL-18, IL-6, TNF-α, and C-reactive protein), inhibition of foam cell formation, and atherosclerotic plaque stabilization [20,21]. Colchicine is also associated with the suppression of vascular endothelial growth factor, leading to a reduction in endothelial proliferation and fibrosis [19]. A study postulated that using this medication before radiation therapy could inhibit microtubule polymers necessary for the acute phase reactants before the acute phase of inflammation occurs, and, thus, attenuate the damage caused by radiation to the cardiovascular system [18]. Other drugs that have shown potential in preventing radiation-induced heart diseases are statins, angiotensin-converting enzyme inhibitors, transforming growth factor-beta 1 inhibitors, recombinant human neuregulin-1, metformin, and aspirin [15,22]. However, their possible role in primary prevention should be further investigated.

## Conclusions

Radiation therapy is not a well-known cause of PAD among medical providers. As a result, this case report aims to encourage others to report similar occurrences and further investigate how to prevent and treat endothelial cell damage. Healthcare providers should be aware that radiation therapy can cause PAD, and patients should be screened for symptoms of PAD regularly and managed appropriately as soon as symptoms appear to prevent the rapid progression of the disease and complications.
